# Remote Measurements of Heart and Respiration Rates for Telemedicine

**DOI:** 10.1371/journal.pone.0071384

**Published:** 2013-10-08

**Authors:** Fang Zhao, Meng Li, Yi Qian, Joe Z. Tsien

**Affiliations:** 1 Brain and Behavior Discovery Institute and Department of Neurology, Georgia Regents University, Augusta, Georgia, United States of America; 2 Yunnan Banna Primate Model Research Center, BanNa Biomedical Research Institute, XiShuangBanNa, Yunnan, China; Rutgers University, United States of America

## Abstract

Non-contact and low-cost measurements of heart and respiration rates are highly desirable for telemedicine. Here, we describe a novel technique to extract blood volume pulse and respiratory wave from a single channel images captured by a video camera for both day and night conditions. The principle of our technique is to uncover the temporal dynamics of heart beat and breathing rate through delay-coordinate transformation and independent component analysis-based deconstruction of the single channel images. Our method further achieves robust elimination of false positives via applying ratio-variation probability distributions filtering approaches. Moreover, it enables a much needed low-cost means for preventing sudden infant death syndrome in new born infants and detecting stroke and heart attack in elderly population in home environments. This noncontact-based method can also be applied to a variety of animal model organisms for biomedical research.

## Introduction

The accurate measurement and monitoring of physiological parameters, including blood volume pulse (BVP), heart rate (HR), respiratory wave (RW), and respiration rate (RR), plays an important role in a wide variety of applications in healthcare, psycho-physiological (polygraph) examinations, sports training, and laboratory animal research [1]–[4]. Dynamic changes in physiological parameters can reveal changes in the physiological status and function of a patient or laboratory animals [4]. In addition, since apnea (abrupt stopping of respiration) and bradycardia (rapid decrease of heart rate) are parts of the final pathway resulting sudden infant death syndrome (SIDS), the most devastating cause of death in infants [5], [6], monitoring dynamic changes of physiological parameters is also important for neonatal care in home environments.

Traditional techniques for the measurement of physiological parameters require sensors to be attached to a subject, such as electrocardiogram (ECG), pulse oximetry, piezoelectric transducer, respiratory-effort belt transducer, and so on. These kinds of contact-based methods may cause undesirable skin irritation, discomfort and soreness to the subject to be measured, especially for neonate [7], [8]. In particular, it may be undesirable to affix sensors to patients during sleep studies (when they may influence a subject's sleep patterns) or during sports training (when they may adversely influence an athlete's mobility). In some cases, contact-based measurements have also been shown to influence the underlying physiological parameters being measured. Currently, Laser Doppler [9], Microwave Doppler Radar [10], Ultra-Wideband Radar [11], [12] and Thermal Imaging [13] have been investigated for the contact-free measurement of physiological parameters with varying success; however, all of these systems require expensive and specialized hardware. As a result, there is a great interest in low-cost and convenient non-contact methods of measuring and monitoring physiological signals.

Recent advances in video technology and machine vision technology [14]–[16] have allowed camera/camcorders to become an indispensable part of both telemedicine/telehealthcare and laboratory animal research. These cameras have been used for object detection, fall detection and posture recognition in elderly healthcare [17], [18], dermatology diagnosis in teledermatology [19], [20], or behavior studies in animal research [1]–[3], . The ability to measure physiological parameters, including HR and RR, using a video camera has been recently reported [23]–[27]. Though attractive in principle, many of these methods accomplish noise reduction using linear filters, which are ineffective in the event that background noise falls within the same frequency band as the physiological signal of interest. Others have proposed using blind source separation for noise removal (Poh, et al.) [28], [29]. Yet, blind source separation methods require a multi-channel signal input, restricting its applicability to process signals collected using a camera which must provide a multi-channel signal (such as a color camera that generates a Red, Green, and Blue multiple-channel signal). As a result, these methods cannot be used to determine physiological parameters in conditions when ambient light is insufficient to permit the use of a color camera, such as in a darkened room or at night when patients or animals need to sleep. Moreover, blind source separation does not provide a critical method to distinguish false positives from actual results. As such, it will frequently generate false physiological parameters when an inanimate object, such as a drawing or picture of a human face, is imaged. These limitations would greatly hamper the application of blind source separation methods in many healthcare situations (during the night when the monitoring is most needed), as it cannot reliably indicate a loss of vital signs in a subject (i.e. sudden death in infants during the neonatal care period at home).

Therefore, we set out to develop a novel method capable of non-contact measuring one or more physiological parameters, including blood volume pulse, heart rate, respiratory wave, and respiration rate, in a subject, particularly at night. Moreover, we set a stringent criterion that such a method should be capable of distinguishing false positives from actual results. In this paper, we describe this novel method to extract heart rate and respiratory rate from single channel images, based on a combination of images-based technologies and a non-linear dynamical systems framework. We also explain how to use the ratio-variation PDs for removing false signals. The applications to humans and several animal models are also presented.

## Materials and Methods

### Ethics statement

Measurement on humans was approved by the Research Ethics Board at BanNa Biomedical Research Institute (BBRI) and all participants provide their written informed consent to participate in this study. All animal work described in the study was conducted in accordance with the National Institutes of Health guidelines, and approved by the Institutional Animal Care and Use Committee of Georgia Regents University.

### System set-up

The image acquisition device is a near-IR enhanced camera (Aptina Imaging Corporation models MT9V024) that is sensitive to light in the visible and near infrared region (between about 400–1,000 nm). Indoor ambient light and a near infrared light emitting diode (LED, 830 nm) served as a source of illumination for daytime and nighttime measurements, respectively. The images are captured in a single channel (8 bits, 0–255) at regular time intervals by a PC (T7500, Dell) over a period of time between three seconds and several minutes. Typically, the distance between camera and subjects is 0.5–3.0 m.

### Reconstruction of the dynamic system

The physiological signals of interest in this paper are the cardiovascular pulse, which is also called the blood volume pulse (BVP), and respiratory wave (RW). The changes of blood volume during the cardiac cycle modify the amount of light absorption in the blood vessel, and thus modulating the reflection amount of the illumination source. The body surface movements caused by respiration modify the path length of the illumination light, and the subsequent changes of the reflected light indicate the timing of respiration events. By capturing the images with camera, the image sensors collect the reflected light signal along with noise due to artifacts. As a result, the corresponding variations in brightness of the skin area and the chest/abdomen area where moved due to breathing indicate the cardiovascular events and respiratory events, respectively. Thus, a single observed signal for each physiological signal to be measured can be formed over time from a series of captured images.

The cardiovascular and cardiorespiratory autonomic system is dynamic in nature. The dynamics of the system are similar to those of other deterministic systems showing chaotic properties [30], which have irregular periodicities as well as an exquisite sensitivity to the initial conditions. To describe the nonlinear dynamics, the state space needs to be reconstructed based on the time-delay embedding theorem [31]–[33], which establishes that it is possible to reconstruct a state space that is equivalent to the original (unobservable) state space composed of all the dynamic variables from only a single observed signal.

Let *τ*_s_ be the sampling time of observation that is equal to the reciprocal of frame rate of the image acquisition device *f*_s_. The lag/delay vector can be written as follows:
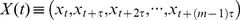
(1)where *τ*  =  *dτ*_s_ is the time delay, *m* is the embedding dimension. A minimal requirement is that m must be as large as the dimension of the attractor of the system.

An embedding matrix can be constructed out of a number of consecutive delay vectors as follows:
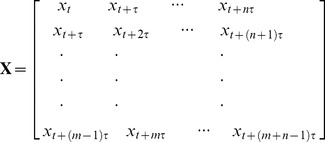
(2)


In practice, the number of delay vectors *n* is determined by the length of the observed signal to be analyzed; however, the number of delay vectors must be at least as large as one physiological signal.

### Physiological signal separation

The embedding matrix contains the physiological information in a mixed form which therefore needs to be deconstructed. ICA is utilized to perform on the embedding matrix to uncover the underlying source signals in the embedding matrix. ICA assumes that the observed signal is a linear mixture of the underlying source signals that can be expressed as
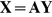
(3)where **Y** is an *m*×*n* matrix containing the independent source signals, **A** is the *m*×*m* mixing matrix, **X** is an *m*×*n* matrix containing the observed signals, which denotes the embedding matrix herein. The aim of ICA is to find a separating or de-mixing matrix **W** such that
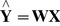
(4)is an estimate of the vector Y containing the underlying source signals.

Any suitable ICA algorithm may be utilized [34]–[39]. In this paper, Fast ICA is used because of both its ease and high speed of implementation [37]–[39]. Fast ICA attempts to obtain **W** by maximize the non-Gaussianity nature of each source. In practice, the fast iterative methods are undertaken to get projections that maximize the Kurtosis (fourth order cumulate).

### False signal identification

When an inanimate object, such as a drawing/picture of a human face or a human being absence of vital signs, is imaged, the signal recovered from images is a false signal, which needs to be identified so as not to generate false positive.

The ratio-variation PDs are the probability distributions of the variation of peak power density ratio for the source signal before and after smooth filtering, where the peak power density ratio is the ratio of highest to total power within the power spectrum of the source signal. The total power in here was calculated by adding up the power within the frequency range of 0∼8 Hz. The variation of the peak power density ratio is defined as follows:
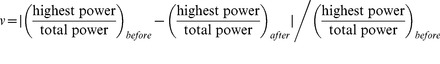
(5)


A living being suggests the existence of a dominant peak in the power spectrum of the source signal that corresponds to the dominancy of the periodic rhythm of physiological signal within a short time window. Meanwhile, there is no generator underlying the observed signal when imaging an inanimate object, thus the source signal consists of random noise with wideband power spectrum. It is conceivable that the variation of peak power density ratio before and after smooth filtering for the true signals should be much smaller than for the false signals. Therefore, it can be supposed that ratio-variation PDs of real and false subjects are distinguishable.

The ratio-variation PDs of live human subjects and inanimate human-shaped figures were estimated from a set of measurements, respectively. Then, the source signal can be identified as true or false according to which ratio-variation PDs of these two distributions is bigger. Photographs of humans in magazines, drawings of a human face and animated characters were used as fake figures.

### Measurement methodology

**Figure 1:**
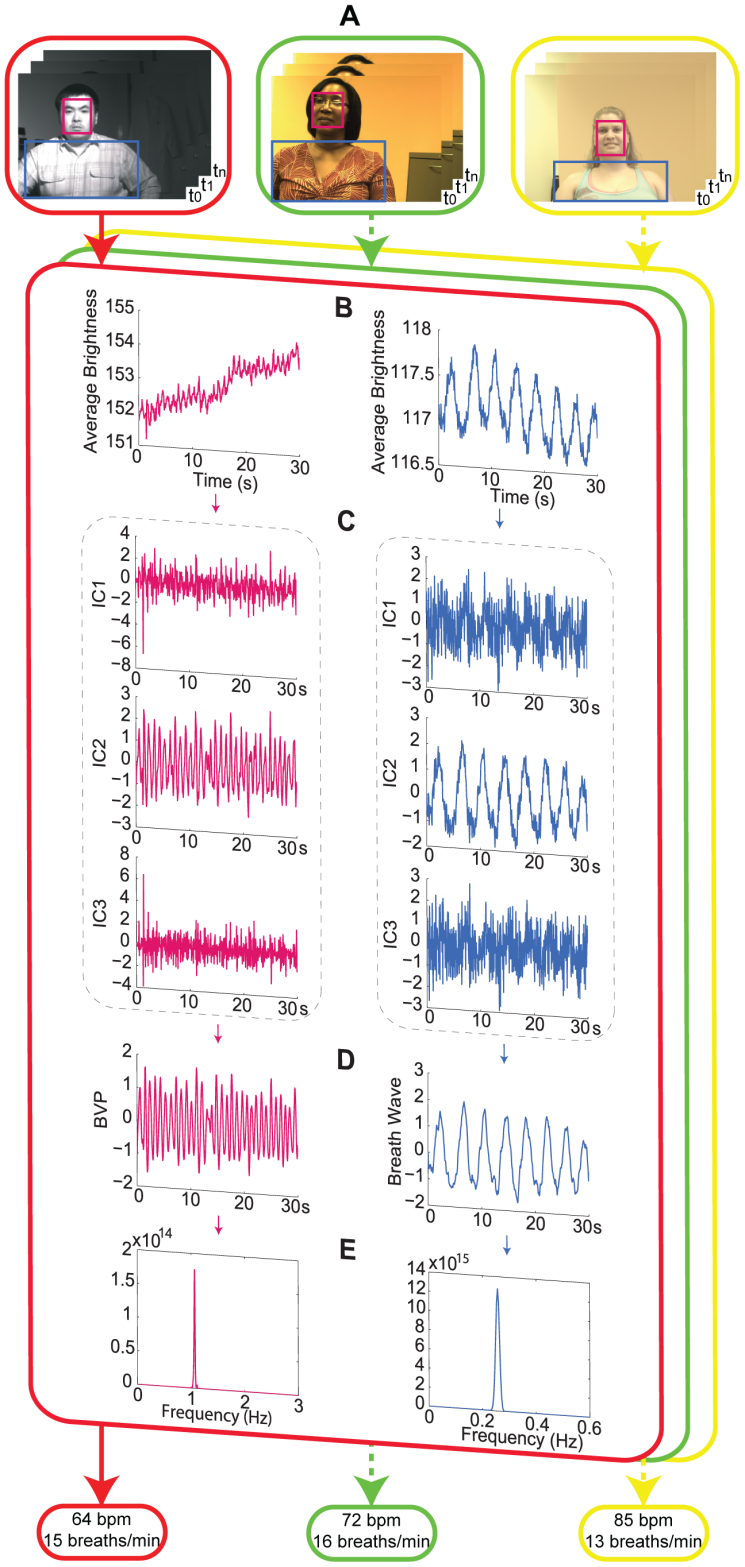
Remotely obtain physiological parameters from single channel images. Different colors of the rounded rectangles in the figure denote the procedure of different subjects. (A) A series of video frames of the upper body of a human subject recorded at night over a period of time (*t*_0_–*t*_n_). The rectangle superimposed on the subject's face indicates the region of interest for cardiovascular pulse measurement. The rectangle superimposed on the subject's chest indicates the region of interest for respiratory measurement. (B) The observed time series for cardiovascular pulse measurement (left) and respiratory measurement (right). (C) The separated source signals (independent components (ICs), IC1, IC2, and IC3) for cardiovascular pulse measurement (left) and respiratory measurement (right). (D) The recovered blood volume pulse (left) and respiratory wave (right). (E) A plot showing the frequency of the blood volume pulse (left) and respiratory wave (right). Written consent from all individuals whose photos are in figures 1 had been obtained.

The general strategy employed for data collection and analysis is generally described in Figure 1. First, subjects were continuous filmed to obtain a series of images over time. Next, the images were analyzed to identify two regions of interest (ROIs) within the image series: one ROI used to determine blood volume pulse and heart rate and a second ROI used to determine respiratory wave and respiration rate (Figure 1A). The ROI can be manually or automatically identified within the images. When the subject to be measured is a human being, we utilize face and upper body detector provided by OpenCV library to automatically find the face and upper body positions of the subject. The detector used by OpenCV is based on Haar cascade classifiers presented by Paul Viloa and Michael Jones [14], as well as Lienhart and Maydt [15]. In our application, the upper body detector is utilized to detect the upper body within the images. The detected regions are then passed on to the face detector to detect faces. We select the center 80% height and 60% width of the face region returned by the detector to be the ROI for cardiovascular pulse measurement, and the area between the bottom of face region and upper body region with a width equal to 80% of the width of upper body region as the ROI for respiratory wave measurement.

Either output channels of the camera can be chosen for physiological signal recovery. Considering the fact that hemoglobin absorptivity is highest in green/yellow light, the Green channel should be a better choice. A measurement point for each physiological signal is obtained from each image within the series of images by spatially averaging the brightness of the pixels from green channel in the ROI. The average brightness obtained for each ROI in a series of images over time are then combined to yield a single observed time series *x*(*t*) (Figure 1B). The obtained single observed time series is detrended using the smoothness prior approach [40], where suitable detrending parameters *λ* can be selected in view of the frequency characteristic and sampling rate of the physiological parameter being measured, and then normalized as 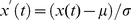, where *µ* and *σ* are the mean and standard deviation of *x*(*t*), respectively.

Using the delay reconstruction, the normalized observed time series were then constructing an embedding matrix with a delay of *d* = 1 and *m* = 3. Fast independent component analysis (ICA) was then performed to decompose embedding matrix into three independent source signals (Figure 1C). The separated component whose power spectrum contained the highest ratio of peak to total energy was then selected to obtain a value for the physiological parameter of interest.

The selected source signal was smoothed using a moving average filter to obtain cardiovascular pulse wave and respiratory wave (Figure 1D). Three-layer autocorrelation was then performed to reduce the residual noise. Finally, a fast Fourier transform (FFT) was performed on the selected source signal to obtain the frequency spectrum of each physiological signal. The frequency of physiological parameters was designated as the frequency that corresponded to the highest power of the spectrum (Figure 1E).

The false signal can subsequently be removed using the ratio-variation PDs of live human subjects and inanimate human-shaped figures according to which of them is bigger.

### Experiment description

Fifteen subjects (7 males, 8 females) with different races and skin color (Caucasians, African Americans, and Asians) between ages of 27–50 years participated in the experiments. Indoor ambient light and a near-infrared LED (830 nm) served as a source of illumination for the daytime and nighttime tests, respectively. Subjects were seated in front of a night vision camera (model MT9V024 available from Aptina Imaging Corporation) at a distance of approximately 1 m to restrict their upper body within the visual field of the camera. Subjects were continuously filmed for a period of three minutes at 15 frames per second (fps) with a pixel resolution of 640×480 and saved in AVI format. During image capture, subjects were instructed to face the camera, remaining seated, and breathe normally. Subjects were allowed to move naturally within a small range, such as looking up/down, nodding, tilting the head or making some facial expressions, but to avoid quick or large motions. Real-time human data acquisition and processing were realized by software written in Visual C++. During the period of image capture, electrocardiography (ECG) and respiratory signals were collected using an OmniPlex® data acquisition system (Plexon, Inc.) via ECG limb electrodes and polyvinylidene difluoride (PVDF) sensor at a sampling rate of 1 kHz, respectively. During the measurement, subjects kept wearing limb electrodes (positive electrode on the left arm and negative electrode on the right foot) to measure the ECG signal, and the PVDF sensor was put under the subject's nose to measure airflow from noise breathing. The ECG and respiratory signals were recorded using Plexon Sort Client software provided by Plexon Inc.

In addition, one of the participants first performed moderate exercise (50 push-ups), and then was seated in front of the camera at a distance of approximately 1 m to restrict their upper body within the visual angle of camera. The subject was continuously filmed for a period of five minutes to measure the dynamic changes of heart rate and respiration rate. During image capture, the subject was instructed to face the camera, remaining seated, and breathe normally. During the period of image capture, electrocardiography (ECG) and respiratory signals were also collected using an OmniPlex® data acquisition system (Plexon, Inc.) at a sampling rate of 1 kHz.

### Measurement of heart rates in mice

A high speed camera (Stingray F-033B/C with 80fps) was used to capture images of a mouse at rest (after running around for about five minutes) over a period of about 10 seconds. The mouse was C57BL6/J mice, and aged 6 months. The ambient light was used as the only source of illumination. During the period of image capture, ECG of the mouse was recorded simultaneously using an OmniPlex® data acquisition system (Plexon, Inc.) via a single pair of insulated electrode wires, which were placed subcutaneously from the back of the neck to the chest. The tips of electrode wires were welded to small stainless springs and then tied to chest muscles. The positive electrode was placed in the lower left part of the chest, and the negative electrode was placed in the upper right side of the chest. The electrode wires were then connected to the OmniPlex® data acquisition system, where ECG signals (filtered at 0.7–300 Hz, digitized at 5 kHz) were recorded using Plexon Sort Client software.

### Measurement of heart rate in zebrafish

Adult zebrafish (roy/roy; alb/alb) were anesthetized using tricaine (MS-222) at 750uM for about 3 seconds, and then the images were captured at 15 fps over a period of about 30 seconds in length. The ambient light was used as illumination source.

### Statistical analysis

**Figure 2:**
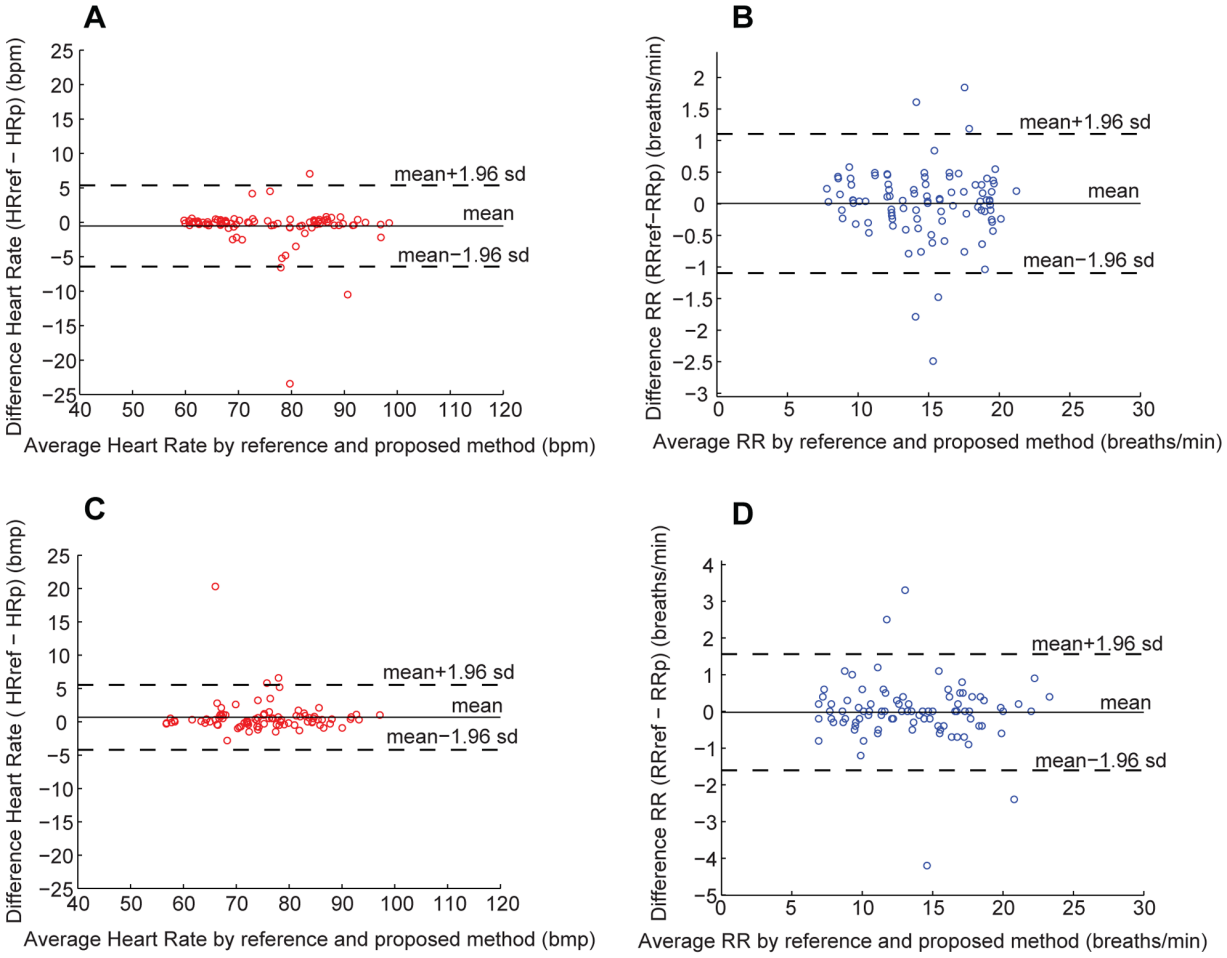
Bland-Altman plots comparing the values obtained for physiological parameters of interest using the presented methods to values obtained using a contact reference method in nighttime and daytime, respectively. The solid horizontal line indicates the mean for the data set; the dotted lines indicate the mean ±1.96 standard deviations. (A) HR of the subject imaged in night conditions. (B) RR of the subject imaged in night conditions. (C) HR of the subject imaged in daytime. (D) RR of the subject imaged in daytime.

Comparison of the presented method to the referenced method was performed using Bland-Altman analysis [41]. The differences between measurements made via image analysis and measurements made using an OmniPlex® data acquisition system (Plexon, Inc.) were plotted against the averages of both systems (Fig. 2). We summarized the degree of agreement by calculating the mean difference 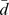 and the standard deviation (SD) of the differences, 95% confidence interval (±1.96 SD), the root mean square error (RMSE), correlation coefficients and the corresponding p-values.

Kolmogorov-Smirnov test [42] was used to estimate the ratio-variation PDs of live human subjects and inanimate human-shaped figures from a set of measurements. Moreover, the false positive rate was calculated as the total number of cases in which an inanimate human-shaped object was incorrectly identified as a human subject divided by the total number of inanimate human-shaped objects measured. The false negative rate was calculated as the total number of cases in which a human subject was incorrectly identified as an inanimate human-shaped object divided by the total number of human subjects measured.

## Results

### Simultaneous measurement of HR and RR in both daylight and night conditions

An example of extracting HR and RR from image sequences of a subject captured at 15 frames per second (fps) with a pixel resolution of 640×480 was illustrated (Figure 1). The Open Computer Vision (OpenCV) library was utilized to automatically identify the region of interest (ROI) for each physiological parameter. The ROIs are outlined by rectangles (Figure 1A). The average brightness in the ROI was calculated for each frame to form a single observed time series over time from recorded frames (Figure 1B). The processing time window was half a minute long. The observed time series was then detrended using a smoothness priors approach [40]. The detrending parameters *λ* = 20 and *λ* = 300 were selected for cardiovascular pulse measurement and respiratory measurement, respectively. Each observed time series was then normalized.

Using the delay reconstruction, the normalized observed time series were then constructed using an embedding matrix with a delay of *d* = 1 and embedding dimension of *m* = 3. Fast ICA was then performed to decompose embedding matrix into three independent source signals (Figure 1C). The separated component whose power spectrum contained the highest ratio of peak to total energy was then selected for further analysis. The selected source signal was smoothed using a moving average filter (five-point for cardiovascular pulse and thirteen-point for respiration) to obtain cardiovascular pulse wave and respiratory wave (Figure 1D). Three-layer autocorrelation and fast Fourier transform (FFT) were performed on the selected source signal to obtain the frequency spectrum. The frequency of physiological parameters was designated as the frequency that corresponded to the highest power of the spectrum (Figure 1E).

The degree of agreement between 95 pairs of measurements for HR and RR from 15 subjects, measured by the method described above and by an OmniPlex® data acquisition system (Plexon, Inc.), was determined by Bland-Altman analysis [41]. The differences between measurements made via image analysis and measurements made using a reference system were plotted against the averages of both systems (Figure 2). For night measurement, the mean difference 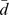 for HR was −0.54 bpm with 95% confidence interval −6.56 to 5.48 bpm (Figure 2A), the root mean square error (RMSE) was 3.13 bpm and the correlation coefficient *r* was 0.96 (p<0.001). For night measurement of the RR, the mean difference 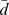 was 0.01 breaths/min with 95% confidence interval −1.12 to 1.13 breaths/min (Figure 2B), the root mean square error (RMSE) was 0.06 breaths/min and the correlation coefficient *r* was 0.99 (p<0.001). For daytime measurements, the mean difference 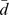 for HR was 0.68 bpm with 95% confidence interval −4.18 to 5.54bpm (Figure 2C), the root mean square error (RMSE) was 3.10 bpm and the correlation coefficient *r* was 0.95 (p<0.001). For daytime measurements of the respiratory rate, the mean difference 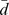 was −0.02 breaths/min with 95% confidence interval −1.69 to 1.65 breaths/min (Figure 2D), the root mean square error (RMSE) was 0.09 breaths/min and the correlation coefficient *r* was 0.98 (p<0.001).

### Dynamic measurement of HR and RR in a subject

**Figure 3:**
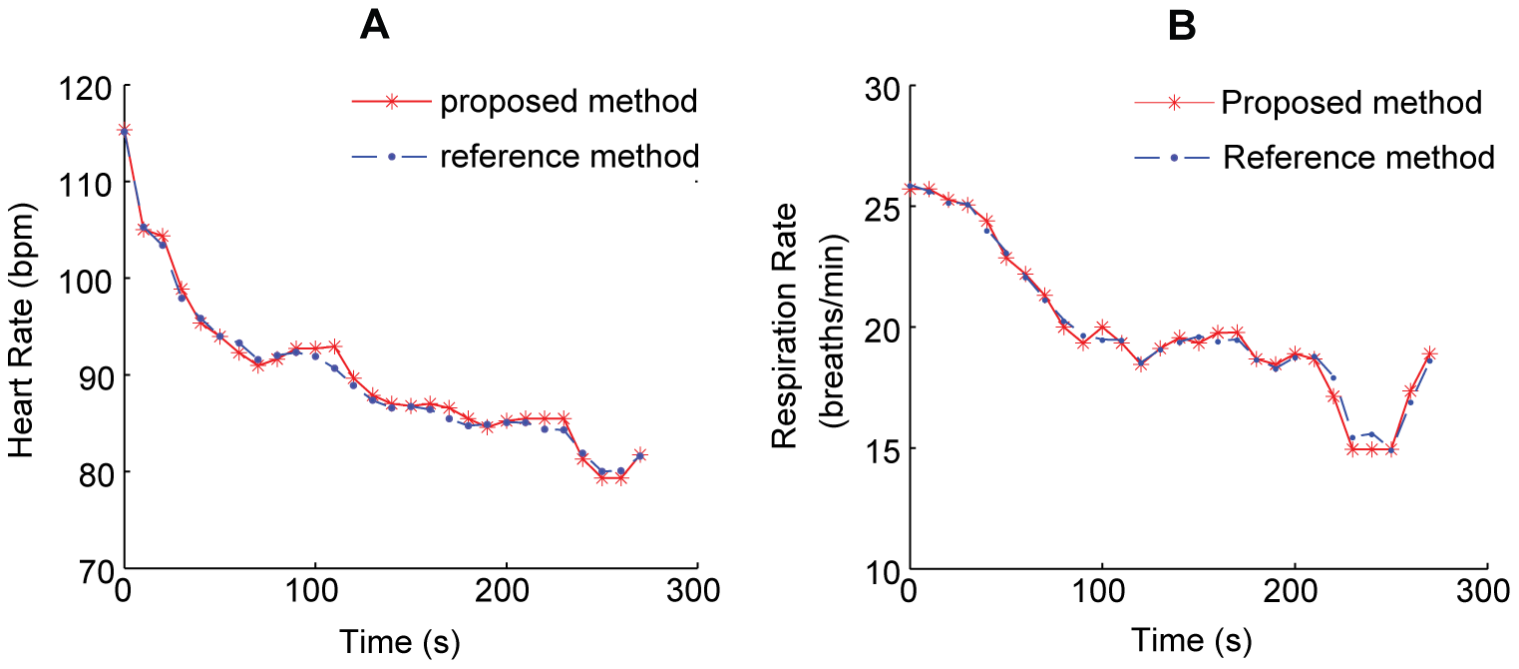
Dynamic measurement of both HR and RR. (A) A plot of HR measured in a subject as a function of time. (B) A plot of RR measured in a subject as a function of time. In both cases, physiological parameters were measured in a subject following moderate exercise.

Some participants first performed moderate exercise, and then were seated in front of a night vision camera to be continuously filmed for a period of five minutes. Following image capture, the images were analyzed, as described in the previous section, to obtain values for HR and RR. These values were compared to the reference values for both HR and RR obtained using the OmniPlex® data acquisition system. Both HR and RR gradually decrease over time. The curves for both HR over time (Figure 3A) and RR over time (Figure 3B) produced by image analysis closely matched values measured by the reference method throughout the test. The correlation coefficient was *r* = 0.995 for both HR and RR. The RMSE for HR and RR were 0.15 and 0.06 respectively.

### Elimination of false signals

To assess the ability of our method to distinguish between live human beings and inanimate human-shaped objects, both human subjects and inanimate human-shaped figures were imaged as described above. 1000 measurements were made from the captured images, with half of the measurements being obtained from imagery of live human subjects and half of the measurements being obtained from imagery of inanimate human-shaped figures. Photographs of humans in magazines, drawings of a human face and animated characters were used as fake figures.

The ratio-variation PDs of live human subjects and inanimate human-shaped figures were estimated from 500 measurements of them, respectively. Kolmogorov-Smirnov test was performed to verify that true signals are in accord with Gamma distribution with parameter *a* = 1.5335 and *b* = 0.0599, and the probability density function (pdf) is given by
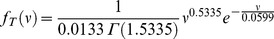
(6)where *ν* represents the variation of peak power density ratio for the source signal before and after smooth filtering.

Meanwhile, the false signals satisfy with Gaussian distribution with mean *µ_o_*  = 0.4905 and standard deviation *σ_o_*  = 0.1434, and the pdf is given by
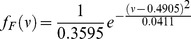
(7)


**Figure 4:**
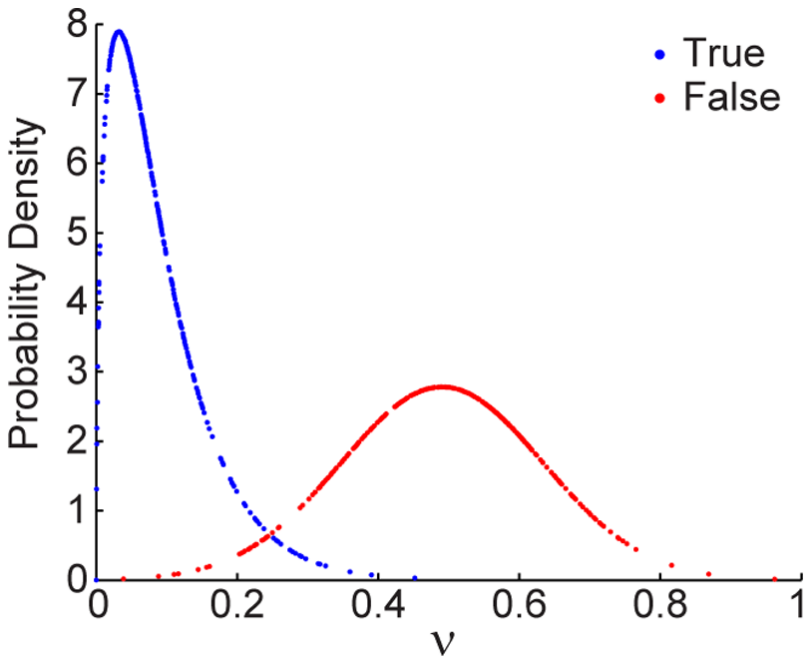
The ratio-variation PDs. Blue points: the ratio-variation PDs of real subjects. Red points: the ratio-variation PDs of inanimate subjects.

**Figure 5:**
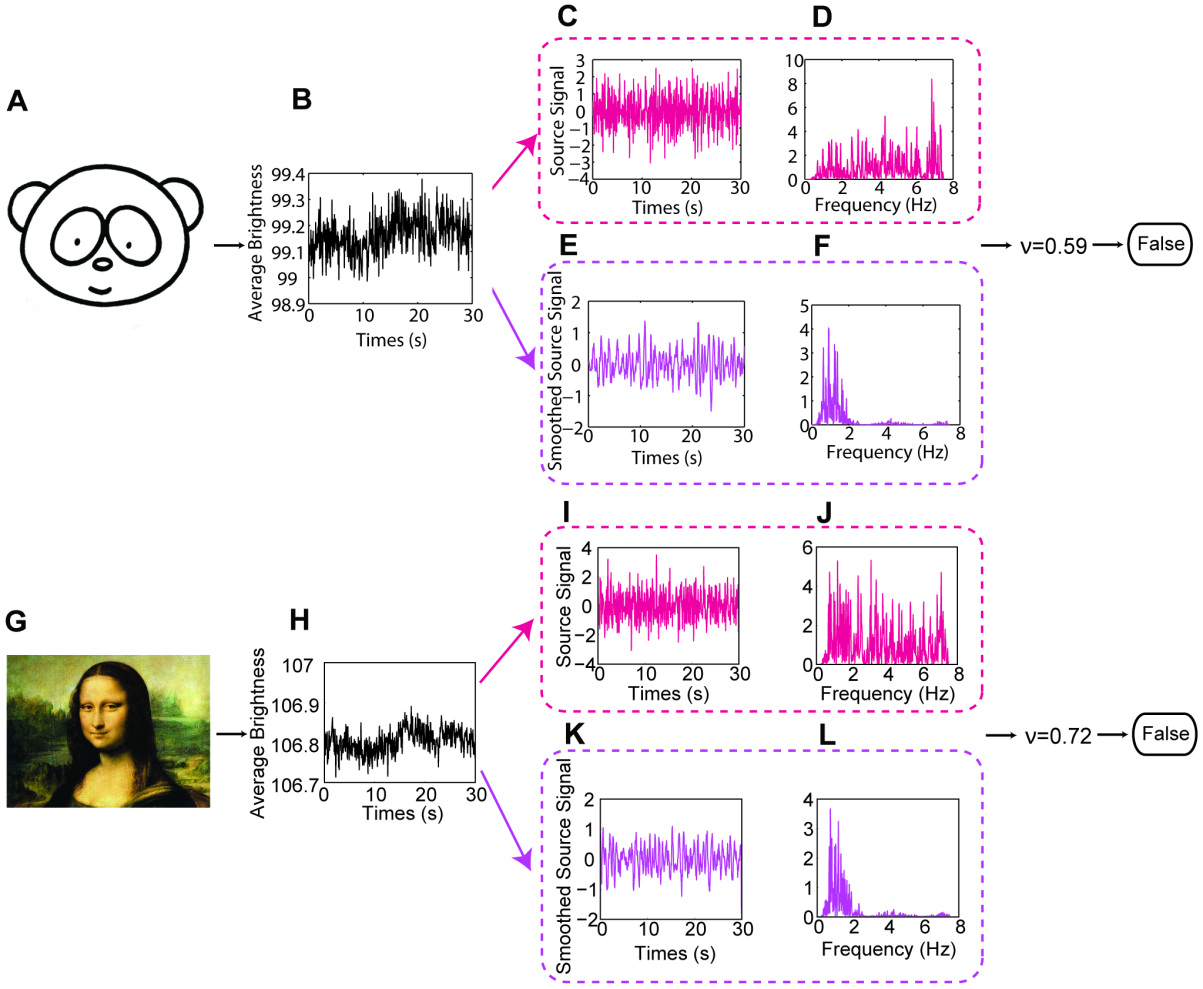
Examples of false signals recognition. (A) Take a Simpsons cartoon character as an example. (B) The observed time series of the Simpsons cartoon is decomposed to extract (C) the source signal and (D) its power spectrum. (E) The smoothed source signal and (F) its power spectrum. (G) Another example is the Mona Lisa painting. (H) The observed time series of the Mona Lisa painting is decomposed to extract (I) the source signal and (J) its power spectrum. (K) The smoothed source signal and (L) its power spectrum.

It can be found that the ratio-variation PDs of real and false subjects are distinguishable (Figure 4). Examples of false signals recognition are shown (Figure 5). Take a Simpsons cartoon character as an example (Figure 5A to F), the variation of peak power density ratio is *ν* = 0.49. According to Eq. (6) and (7), *f_T_*(*ν* = 0.49) = 0.016 is smaller than *f_F_*(*ν* = 0.49) = 2.78, thus the Simpsons character is correctly recognized as an inanimate subject. Another example is the Mona Lisa painting (Figure 5G to L), the variation of peak power density ratio is *ν* = 0.72. According to Eq. (6) and (7), *f_T_*(*ν* = 0.72) = 4.24e–4 is much smaller than *f_F_*(*ν* = 0.72) = 0.77. As a result, the Mona Lisa can be identified as an inanimate figure.

To evaluate the false positive and false negative rates, other 1000 measurements were made, with half of the measurements being obtained from imagery of live human subjects and half of the measurements being obtained from imagery of inanimate human-shaped figures. Overall, the false positive rate was found to be 9.8%, and the false negative rate was found to be 6.5%.

We utilized the sum of the probability of three successive measurements (ten seconds in length, taken 5 s apart) to improve the false rate. The artifacts were rejected by comparing the accumulate probability of the real and false subjects. If the accumulate probability of the false cases exceeds that of the true cases, the subject would be recognized as an inanimate subject, and vice verses. After using this criterion, the false positive rate was found to decrease substantially to 2.3%, and the false negative rate was found to decrease to 0.9%.

### Utility on animals and internet videos

**Figure 6:**
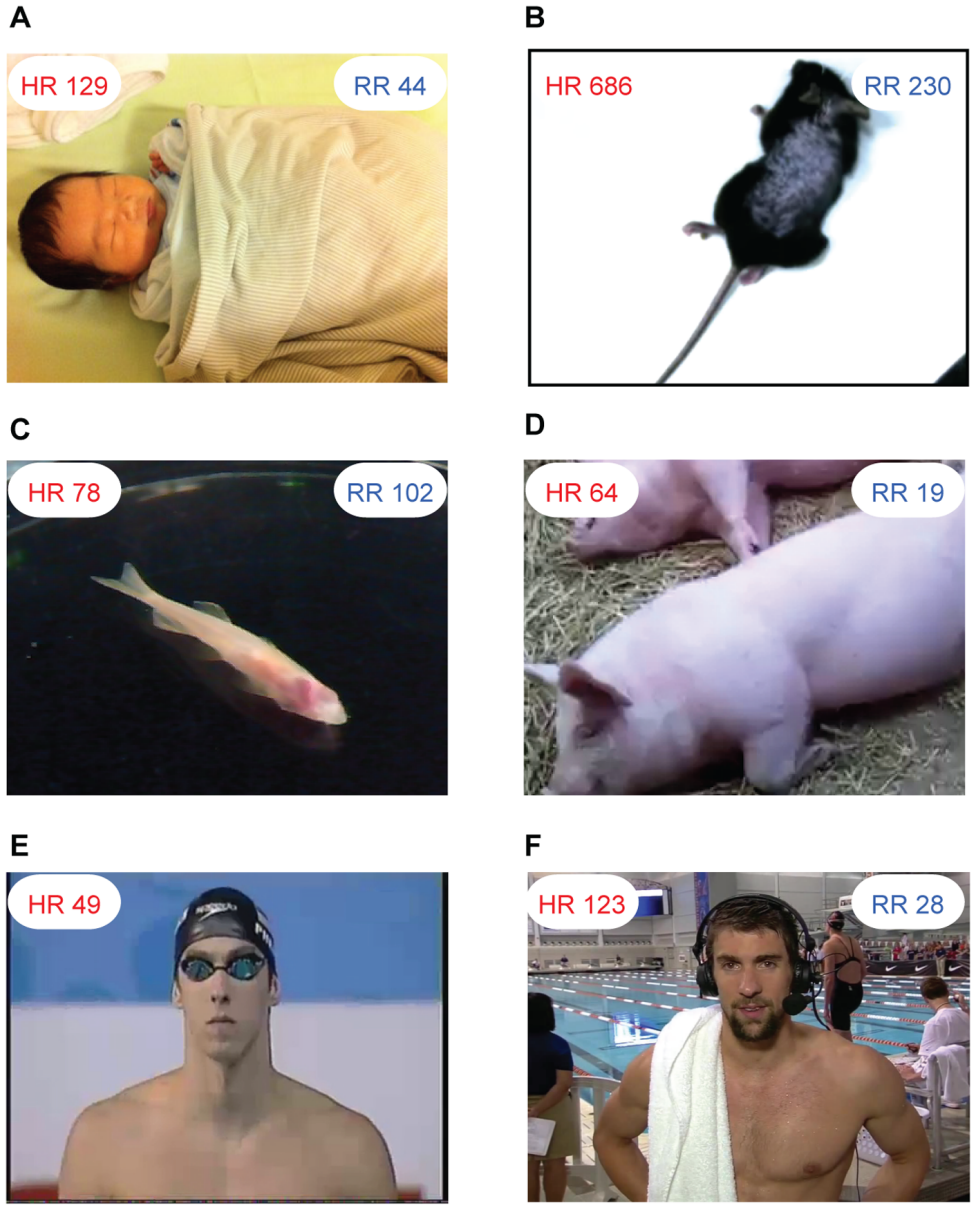
Measurement results for different kinds of subjects. (A) Thirty seconds video of a one-month old infant was recorded at 30fps when he was sleeping. The face area and shoulder area are selected as the ROI for cardiovascular pulse measurement and respiration measurement, respectively (Figure 7A). In the spectrum of the BVP signal (Figure 7H), a clear peak at 2.15 Hz corresponds to the HR of 129 bpm. In the spectrum of the breath wave signal (Figure 7I), an obvious peak at 0.73 Hz corresponds to the RR of 44 breaths/min. Written consent from the child’s parents had been obtained. (B) A high speed camera (Stingray F-033B/C with 80fps) was used to capture images of a mouse at rest over a period of about 10 seconds. Figure 8 demonstrates the procedure of recovering the HR and RR of a mouse from a single channel images. The measurement result of RR is 230 breaths/min and the measurement result of HR is 686 bpm. (C) For measuring adult zebrafish (roy/roy; alb/alb), we used tricaine to slightly anesthetize before the images were captured at 15 fps over a period of about 30 seconds in length. The ROI for cardiovascular pulse measurement and respiration measurement is the heart area and gilles area, respectively (Figure 9A). The measurement result for HR is 78 bpm and for RR is 102 breaths/min (for signal plot, see Figure 9). (D) The face area and abdomen area of the pig are chosen as the ROI for cardiovascular pulse measurement and respiration measurement, respectively (Figure 10A). The measurement results of HR and RR are 64 bpm and 19 breaths/min, respectively (for signal plot, see Figure 10). (E) A video of Phelps before 200 M butterfly competition downloaded from YouTube (http://www.youtube.com/watch?v=ftHlLqamWlM). The recovered HR from 3 seconds time period selected from the video is 49 bpm. (F) Post-race interview of Michael Phelps (after his 200 Fly meet) downloaded from YouTube (http://www.youtube.com/watch?v=HHy7QKEV310). The interview video was recorded at 29 fps with pixel resolution of 1280×720. Two time periods both about 3 seconds in length were selected from the video to recover the HR of Phelps (Figure 11). The time interval between two time periods is within one minute. The measurement results of HR for the first and second time periods are 123bpm and 126bpm, respectively. The RR was also extracted from the video over a period of about 7 seconds. The measurement result of RR is 28 breaths/min (for signal plot, see Figure 12). Reprinted from [43] under a CC BY license, with permission from [Longhorn Network], original copyright [2012]

**Figure 7:**
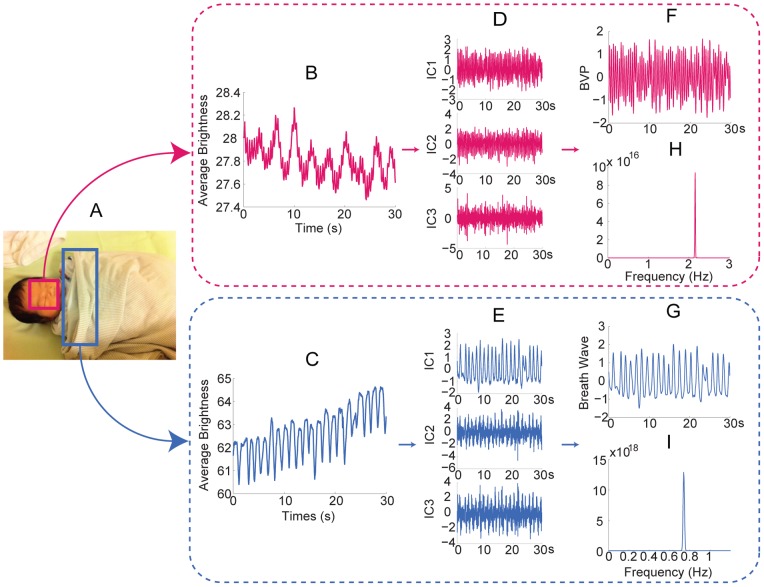
Measurement of neonate. (A) The rectangle superimposed on the neonatal face indicates the ROI for cardiovascular pulse measurement. The rectangle superimposed on the neonatal shoulder indicates the ROI for respiratory measurement. 30 seconds observed time series for cardiovascular pulse measurement (B) and respiratory measurement (C). The separated source signals for cardiovascular pulse measurement (D) and respiratory measurement (E). The recovered blood volume pulse (F) and respiratory wave (G). The spectrums of the blood volume pulse (H) and respiratory wave (I). Written consent from the child's parents had been obtained.

**Figure 8:**
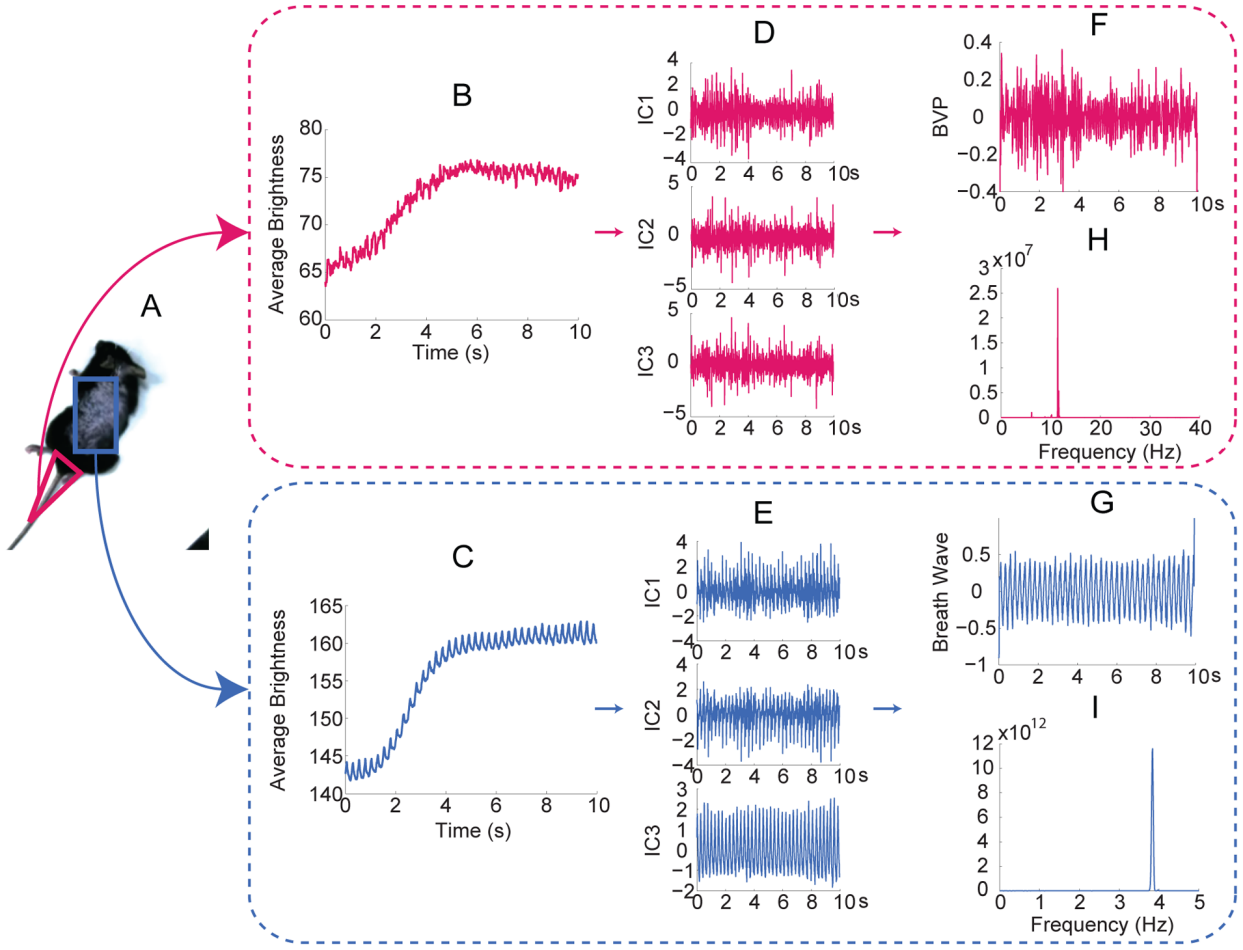
Measurement of a mouse. (A) The triangle and rectangle superimposed on the mouse indicate the ROI for cardiovascular pulse measurement and respiratory measurement, respectively. 10 seconds observed time series for cardiovascular pulse measurement (B) and respiratory measurement (C). The separated source signals for cardiovascular pulse measurement (D) and respiratory measurement (E). The recovered blood volume pulse (F) and respiratory wave (G). The spectrums of the blood volume pulse (H) and respiratory wave (I).

**Figure 9:**
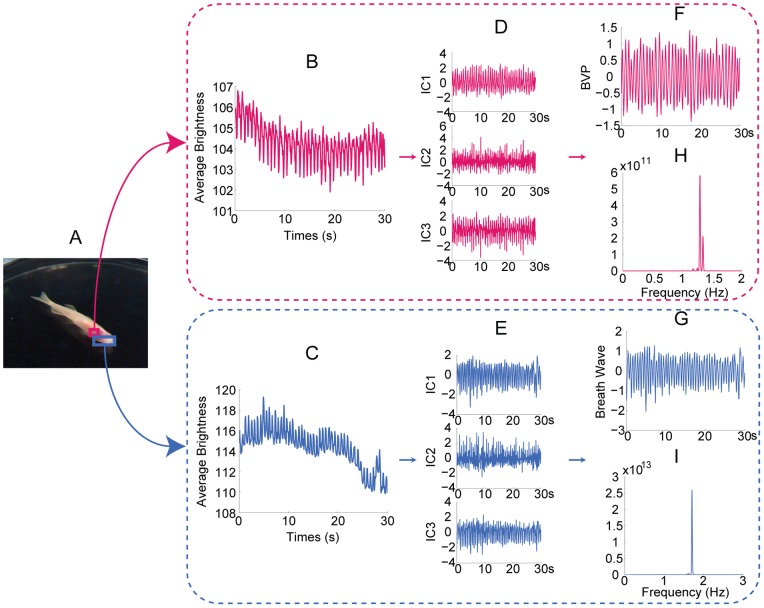
Measurement of a zebrafish. (A) The rose and hyacinthine rectangle superimposed on the zebrafish indicate the ROI for cardiovascular pulse measurement and respiratory measurement, respectively. 30 seconds observed time series for cardiovascular pulse measurement (B) and respiratory measurement (C). The separated source signals for cardiovascular pulse measurement (D) and respiratory measurement (E). The recovered blood volume pulse (F) and respiratory wave (G). The spectrums of the blood volume pulse (H) and respiratory wave (I).

**Figure 10:**
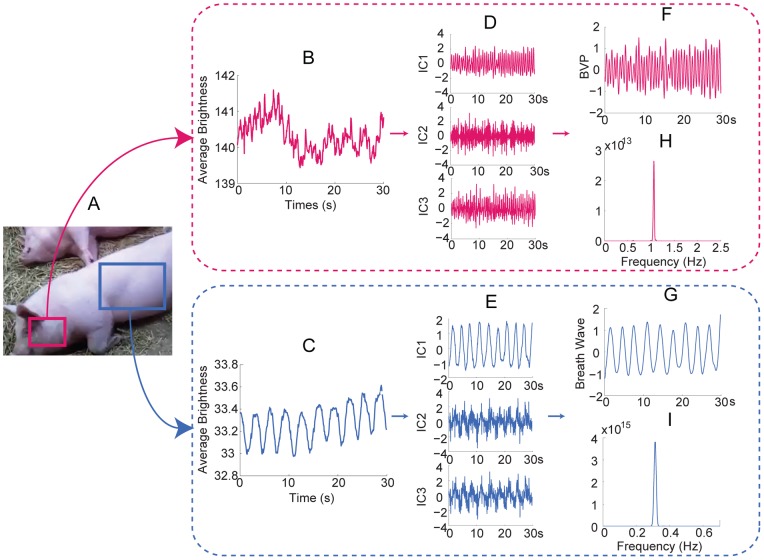
Measurement of a pig. (A) The rose and hyacinthine rectangle superimposed on the pig indicate the ROI for cardiovascular pulse measurement and respiratory measurement, respectively. 30 seconds observed time series for cardiovascular pulse measurement (B) and respiratory measurement (C). The separated source signals for cardiovascular pulse measurement (D) and respiratory measurement (E). The recovered blood volume pulse (F) and respiratory wave (G). The spectrums of the blood volume pulse (H) and respiratory wave (I).

**Figure 11:**
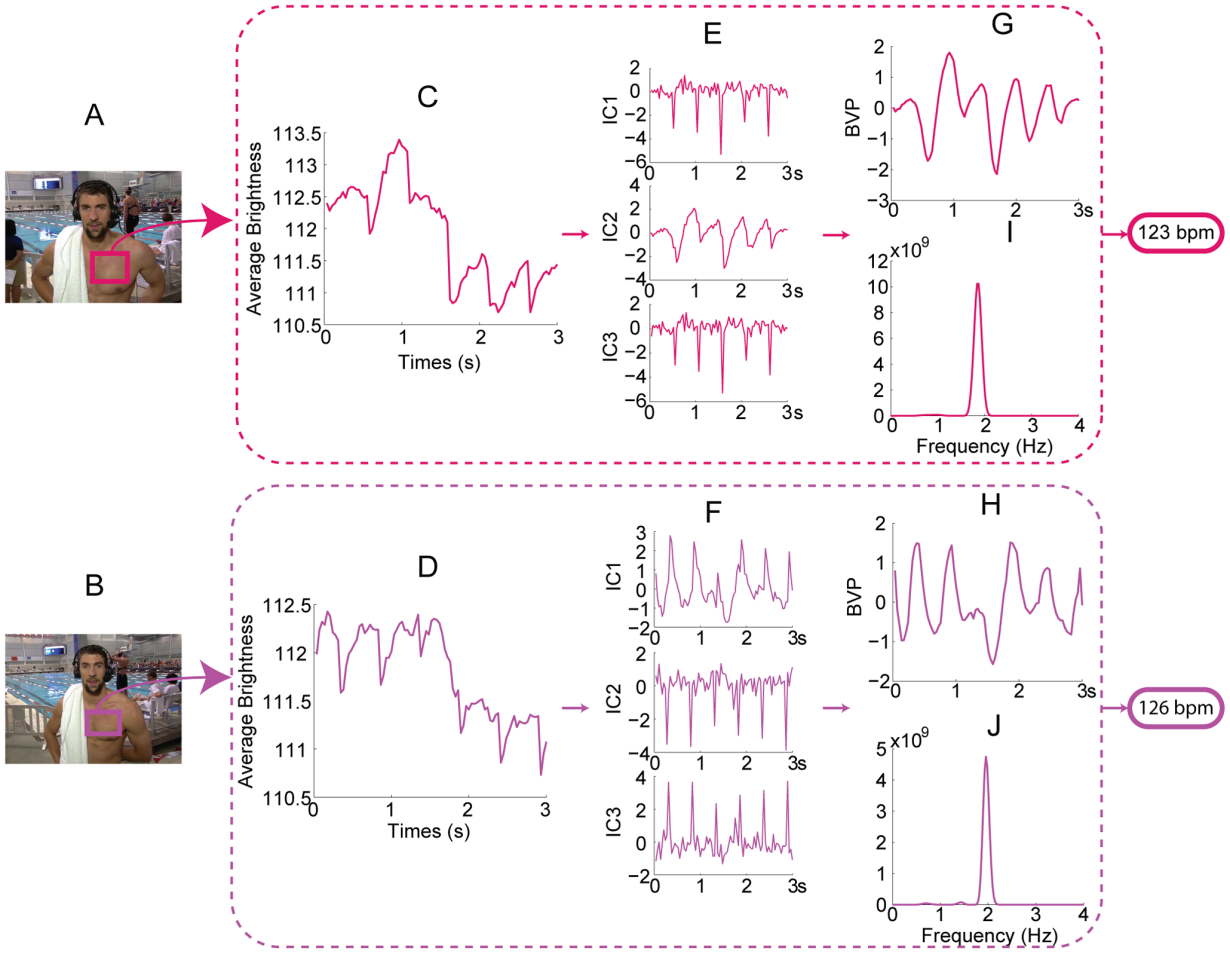
Extracting HR from Michael Phelps video obtained from YouTube. All the red curves correspond to the first time period, and all the purple curves correspond to the second time period. The rectangles superimposed on his chest indicate the ROI for the first time period (A) and the second time period (B). 3 seconds observed time series from the first time period (C) and the second time period (D). The separated source signals for the first time period (E) and the second time period (F). The recovered blood volume pulses for the first time period (G) and the second time period (H). The spectrum of the blood volume pulses for the first time period (I) and the second time period (J). Reprinted from [43] under a CC BY license, with permission from [Longhorn Network], original copyright [2012].

**Figure 12:**
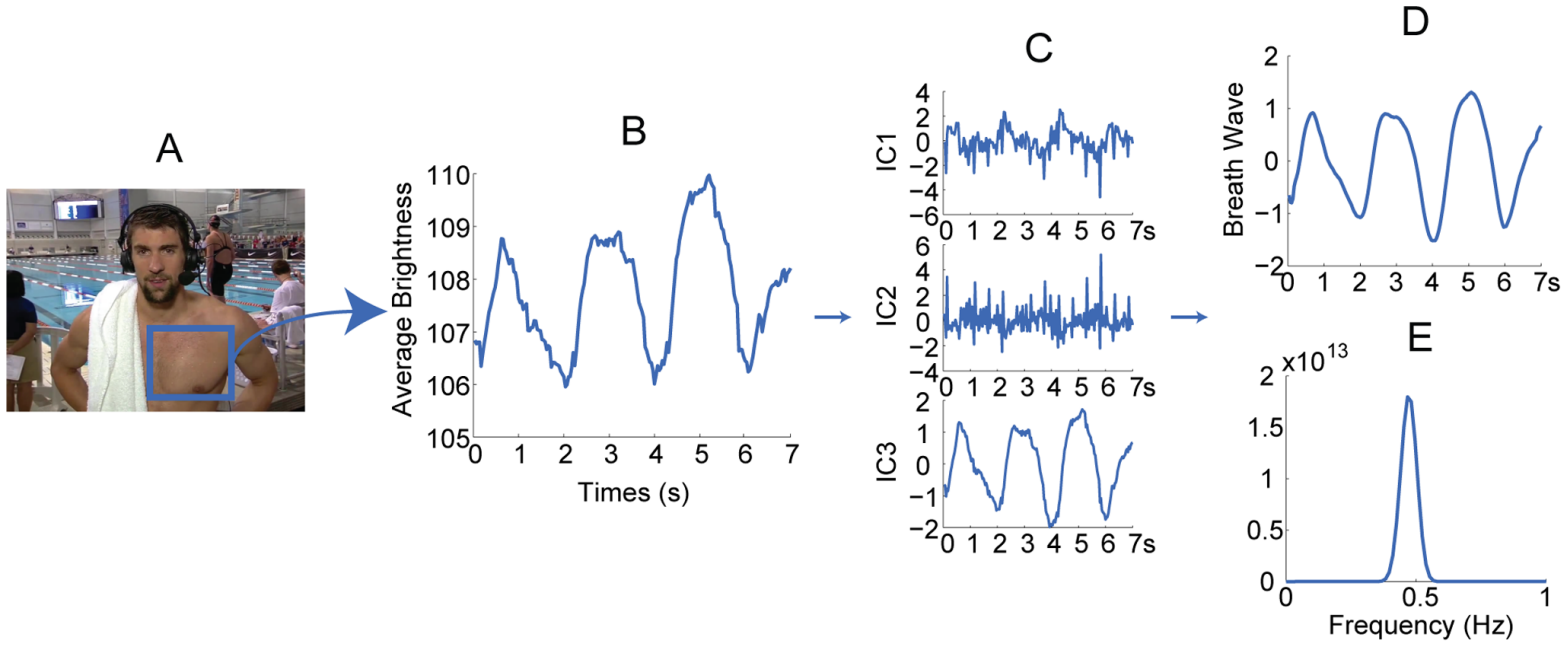
Extracting RR from Michael Phelps post-race interview video obtained from YouTube (http://www.youtube.com/watch?v=HHy7QKEV310). (A) the rectangle superimposed on his chest indicates the ROI. (B) 7 seconds observed time series for respiratory measurement. (C) the separated source signals for respiratory measurement. (D) the recovered breath wave. (E) the frequency of the breath wave. Reprinted from [43] under a CC BY license, with permission from [Longhorn Network], original copyright [2012].

We applied this methodology under a wide variety of situations and the results are shown (Figure 6, for signal processing plot, see Figure 7, 8, 9, 10, 11, 12). For measuring infant (one-month old) (Figure 6A), we can recover the HR and RR of neonate from a series of images from an infant in sleep. The camera was set above his crib and the ambient light was used as the only source of illumination. For obtaining physiological signs from mice in our laboratory (Figure 6B), we used a high speed camera (Stingray F-033B/C with 80fps) to capture images of a mouse at rest (after running around about 5 minutes) over a period of about 10 seconds. Again, only ambient light was used as the illumination source. We found that for measuring HR, the ROI should be the mouse tail and the posterior end of the body. This is because the whole body of the mouse moves as a consequence of respiration which produces very strong signals as such that the BVP signal is lost within it. We highpass filtered (5.8 Hz) the single observed time series formed in ROI of BVP. The HR results measured by the images and by an OmniPlex® data acquisition system are almost the same values. For zebra fish and pigs (Figure 6C and D), we analyzed the images captured at 15 fps over a period of about 30 seconds in length. The cardiovascular pulse measurements from the zebra fish and the pig were 78 bpm and 64 bpm, respectively. In addition, we can also apply our method to a video clip on TV or the internet (i.e. interviews of Michael Phelps after his swimming competition) to obtain vital signs (Figure 6E).

## Discussion

In this present study, we report, for the first time, a novel method for rapid and remote measurement of HR and RR in both day and night conditions. A key feature is that an embedding matrix is taken as a basic description of the cardiovascular and cardiorespiratory autonomic system. The embedding matrix is constructed from a series of delay vectors taken from the observed signal. Judging from the statistical measurements (Table 1), we can achieve high degrees of agreement between values obtained by our method and the reference method even in the presence of motion artifacts. A limitation is that the temporal resolution of the observed time series recorded by camera is not very high due to the low frame rate of the conventional commercially available camera (usually between 15–30fps). Nonetheless, the results obtained in this study have verified the accuracy and effectiveness of this method to extract physiological parameters from a single channel image under such constraint. It is conceivable that various high-speed cameras can be used for more specialized situations.

**Table 1:** Bland-Altman results of HR and RR measurements using presented method and reference method.

Statistic parameters	HR (bpm)		RR (breaths/min)	
	Daytime	Nighttime	Daytime	Nighttime
Measurement pairs	95	95	95	95
Mean difference	0.68	−0.54	−0.02	0.01
Standard deviation of difference	2.48	3.07	0.85	0.57
Upper limit	5.54	5.48	1.65	1.12
Lower limit	−4.18	−6.56	−1.69	−1.13
RMSE	3.10	3.13	0.09	0.06
Correlation coefficient	0.95	0.96	0.98	0.99

Moreover, due to the fact that hemoglobin absorptivity in the IR band is significantly less than that in the visible band (especially for green/yellow wavelength), the IR light provides much lower sensitivity to blood pulsation than ambient light does. Since the auto gain control (AGC) function in most of the conventional cameras (which effectively raises the brightness of the image if the subject becomes darker, and reduces the brightness vice versa) would decrease the dynamics of the signal, we found that switching off the AGC function during nighttime measurement could improve the signal to noise ratio (SNR). As such, the nighttime measurement exhibited a more reliable agreement with the reference method (HR 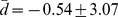
and RR 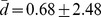). These are comparable with the accuracy achieved by daytime measurement (HR 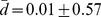 and RR 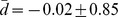).

Although the results demonstrated that our approach can tell false from true, the error rate was not completely satisfactory. The errors were caused by the crossover between the pdfs of the real and inanimate subjects due to random noise. To address this issue, we utilized the sum of the probability of three successive measurements (ten seconds in length, taken 5 s apart) to reject the artifacts by comparing the accumulate probability of the real and false subjects. After using this criterion, the false positive rate was found to decrease substantially to 2.3%, and the false negative rate was found to decrease to 0.9%.

With these approaches, we have shown that it is possible to remotely extract physiological signals from only a single channel images recorded by a camera using dynamic embedding technique followed by ICA. The accuracy and effectiveness of the presented method in detecting vital signs and removing false signals can be achieved in a robust manner. Therefore, the presented method can rapidly indicate changes or a sudden loss of vital signs in an individual, by reliably removing false positive as well as false negative. We believe that this novel, real-time measurement method may have broad applications for remote measurement of vital signs on laboratory animals for biomedical research, or ecological research on wild and endangered animal species. Furthermore, in coupling with emergent sophisticated human action recognition algorithm [16], our method may enable a crucial and low-cost means for early-preventing SIDS in new born infants at home or for detecting stroke or heart attacks in elderly patients at home or nursing homes.
